# Psychosis or Obsessions? Clozapine Associated with Worsening Obsessive-Compulsive Symptoms

**DOI:** 10.1155/2016/2180748

**Published:** 2016-05-30

**Authors:** Jonathan G. Leung, Brian A. Palmer

**Affiliations:** ^1^Department of Hospital Pharmacy Services, Mayo Clinic, 200 First Street, Rochester, MN 55905, USA; ^2^Department of Psychiatry and Psychology, Mayo Clinic, 200 First Street, Rochester, MN 55905, USA

## Abstract

One underrecognized adverse event of clozapine is the emergence or worsening of obsessive-compulsive symptoms (OCS). OCS, particularly violent thoughts, can be inaccurately described as psychosis and result in a misdiagnosis. We report a case of a 42-year-old man, initially diagnosed with schizoaffective, who was placed on clozapine for the management of “violent delusions.” However, clozapine led to a worsening of these violent thoughts resulting in suicidal ideation and hospitalization. After exploration of the intrusive thoughts and noting these to be egodystonic, clearly disturbing, and time consuming, an alternative diagnosis of obsessive-compulsive disorder (OCD) was made. Clozapine was inevitably discontinued resulting in a significant reduction of the intrusive thoughts without emergence of psychosis or adverse events. While an overlapping phenomenology between OCD and psychotic disorders has been described, clozapine and other antiserotonergic antipsychotics have been implicated with the emergence or worsening of OCS. Unique to our case is that the patient's obsessions had been treated as psychosis leading to the inadequate treatment of his primary illness, OCD. This case highlights the potential for OCD to masquerade as a psychotic disorder and reminds clinicians that clozapine may worsen OCS.

## 1. Introduction

Clozapine has superior efficacy compared to other antipsychotics in treatment resistant schizophrenia [1]. It is generally not considered a first-line option due to the risk of agranulocytosis. One adverse event associated with clozapine that occurs in up to 20% of patients is the worsening/emergence of obsessive-compulsive symptoms (OCS) or obsessive-compulsive disorder (OCD) [2]. When OCS from clozapine is interpreted as worsening psychosis, this may lead to a dose increase, further exacerbating symptoms or resulting in declaration of clozapine failure [2, 3]. Several case reports, case series, and small studies support clozapine's association with the worsening/emergence of OCS in patients with a primary psychotic disorder [4–8]. However, data are limited describing this phenomenon in patients with OCD and no comorbid psychotic disorder. We describe a case in which chronic violent and intrusive thoughts led to an initial diagnosis of schizoaffective disorder that was poorly responsive to treatment with antipsychotics. The patient was eventually started on clozapine leading to a further worsening of OCS and hospitalization. We discuss how dose changes and the ultimate discontinuation of clozapine resulted in clinical improvement, allowing for appropriate treatment of the patient's OCD.

## 2. Case Presentation

A 42 year-old man with a past medical history of schizoaffective disorder, hyperlipidemia, and gastrointestinal reflux disease was admitted to an adult psychiatric unit secondary to intrusive thoughts that were sexual, homicidal, and suicidal in nature. Similar thoughts with tics were first reported 2 decades prior to the current hospital admission; however symptoms of depression were most prominent then and he was initially diagnosed with only major depressive disorder. During the following years there were documented reports of “paranoia and delusions related to contamination” as well as continued reports of intrusive violent thoughts also believed to be delusions. This led to a diagnosis of schizoaffective disorder; and antipsychotics became the primary modality of treatment over the next decade. Documented antipsychotics trials included aripiprazole, olanzapine, lurasidone, and quetiapine, all of which were associated with a lack of adequate improvement or adverse events. Records indicated that haloperidol combined with fluvoxamine did result in partial improvement. Antidepressants were also utilized intermittently over this 20-year period to target mood symptoms. Historically, the antidepressant trial with the highest documented relative dose was paroxetine 60 mg. It was during this time period with paroxetine that there was documented improvement of the patient's intrusive thoughts. Side effects limited use of the paroxetine 60 mg, and after it was decreased the violent intrusive thoughts worsened.

Inevitably the patient's outpatient provider after years of suboptimal response to antipsychotics initiated clozapine to target what was continued to be described as psychotic symptoms in the medical record. However, as the clozapine was gradually increased to 400 mg the patient noted a severe worsening of intrusive thoughts causing secondary depressed mood, anxiety, and suicidal ideation leading to hospitalization at our facility. It was during this admission that there was exploration of the patient's intrusive thoughts, which were clearly obsessions in that they were persistent, unwanted, egodystonic thoughts. Efforts by the patient to suppress or ignore these thoughts were futile, resulting in significant anxiety and distress. Yet while these obsessions occupied a significant amount of time during the day, impacting his social and work functioning, he denied any ritualistic behaviors or compulsions. Family history was found to be positive for an aunt and mother with hoarding disorder and a cousin with OCD. As the obsession were not attributable to the use of substances, a medication condition, or other mental disorders, the diagnosis of OCD was given for the first time based on criteria from the Diagnostic and Statistical Manual of Mental Disorders, 5th edition [9]. With the patient now diagnosed with OCD, he was appropriately treated; and there was a plan to initiate fluoxetine with a goal dose of 80 mg. Given the historical nature of the schizoaffective diagnosis, clozapine was continued taking into account there had been improvement during the hospitalization and that it would need to be slowly tapered as an outpatient to avoid adverse effects. The patient was safely discharged with minimal intrusive thoughts and it was recommended to the patient's outpatient provider that clozapine be discontinued after a slow taper. However, in the following months, the patient's outpatient provider had increased clozapine to 700 mg. This was associated again with a subsequent worsening and exacerbation of distressing intrusive thoughts leading to another hospitalization. Serum clozapine and norclozapine levels upon admission were found to be 453 ng/mL and 464 ng/mL, respectively. Clozapine was rapidly discontinued due to the association with OCS, as well as the patient's clinical presentation after each clozapine escalation. Clozapine was tapered over approximately 2 weeks without complications such as cholinergic rebound, catatonia, serotonin syndrome, or withdrawal dyskinesias [10–12]. More importantly there was no emergence of psychotic symptoms, strengthening the likelihood that the patient did not have schizoaffective disorder based on DSM-5 criteria.

In review of records and after discussion with the patient, it was determined that the past fluvoxamine trial did not reach an adequate dose for the treatment of OCD. Fluvoxamine was started and increased to 200 mg after clozapine had been allowed to wash out. Low-dose aripiprazole was also initiated to augment the fluvoxamine given the severity of the presentation, the benefits of this antipsychotic in treatment resistant OCD, and the low risk of inducing OCS [13]. The patient was discharged with resolution of intrusive thoughts and improvement in mood and still without emergence of psychotic symptoms. Following this hospitalization the patient began to see a therapist who specialized in exposure and response prevention therapy to address his OCD. Longitudinally, the patient struggled with depression and anxiety associated with residual obsessions resulting in fluvoxamine being changed to clomipramine. The last known dose of clomipramine was 150 mg and reported as having reasonable control of OCS as an outpatient. Up to a year following the second reported hospitalization at our facility there were no patient reports or documentation noted of criteria sufficient for a diagnosis of schizoaffective disorder or other psychotic disorders.

## 3. Discussion

The presentation of OCD is widely variable among individuals diagnosed with the illness. By definition it is characterized by the presence of obsessions (i.e., recurrent/persistent thoughts, urges, or images that are intrusive and unwanted) that are usually accompanied by compulsions (i.e., the repetitive acts/behaviors conducted in response to the obsession) [9]. Most who suffer from OCD have obsessions and compulsions; but both are not required for the diagnosis. Obsessions and/or compulsions must be time-consuming or cause clinically significant distress. The diagnosis may be further specified by the patient's level of insight into the beliefs and if there is a current or past history of a tic disorder. Affective response to obsessions or compulsions varies among patients with OCD but can invoke anxiety, depressive symptoms, and dysphoria. Avoidance of triggers tied to obsessions and compulsions is common and may significantly impact functioning. Psychotic-like experiences are reported in up to 41% of patients with OCD [14]. This wide variability of symptom presentation can make the diagnosis of OCD challenging. It is estimated that 25% of patient with OCD will attempt suicide and nearly half of patients will report suicidal ideation. This makes the recognition and adequate treatment of OCD crucial in an attempt to reduce morbidity and mortality.

With respect to the relationship between OCS/OCD and psychosis, there is literature discussing OCS/OCD in patients with a primary psychotic disorder; and in fact a “schizo-obsessive” diagnostic entity has been described. It has been estimated that 30% of patients with schizophrenia meet the full diagnostic criteria for OCD [15]. Conversely, literature assessing the psychotic phenomenology associated with purely OCD is less robust [14, 16]. A recent investigation described the characteristics of self-reported hallucinations and delusions in patients with OCD [14]. Authors suggested that psychotic-like experiences can present in patients with OCD, especially in setting of significant emotional distress and anxiety. In our case, the diagnosis of schizoaffective disorder did not clearly fit after understanding the nature of the intrusive thoughts. Other than the intrusive thoughts, initially viewed as delusions, it is not clear if there had ever been a time period of psychosis in the absence of a major mood episode or if any psychotic symptoms were present independent of worsening OCS. The patient's insight to the OCS was intact making delusional disorder also less likely. The patient's recurrent thoughts did not have the real-life concern quality that is associated with generalized anxiety disorder and the avoidance behavior was not associated with hypervigilance or event reexperiencing that is characteristic of posttraumatic stress disorder. By history there were symptoms congruent with the diagnosis of MDD, a common comorbidity with OCD, occurring in up to 41% of patients [17].

The pharmacotherapy aspects of this case are complex and led to several clinical issues related to the management of OCS/OCD and clozapine-induced OCS/OCD. First, we highlight the need for higher antidepressant dosing when treating OCD. In treatment resistant cases and when tolerated, it may not be uncommon to target greater than twice usual maximum doses used in MDD [18]. We note in this case that when higher doses of antidepressants were trialed (i.e., paroxetine 60 mg daily and later fluoxetine to 80 mg) improvement of symptoms was more robust per reports. The patient was also reported to have had benefit from fluvoxamine in combination with haloperidol. The American Psychiatric Association guidelines for OCD recommend augmentation with an antipsychotic in patients who have a partial response to antidepressant therapy, noting modest evidence with haloperidol, risperidone, quetiapine, and olanzapine [18]. Clozapine is not recommended for OCD based on one small open-label trial that failed to demonstrate benefit [19]. Additionally, clozapine has been described to induce or worsen OCS/OCD in mostly small studies and case reports involving patients with a primary psychotic disorder [5, 20, 21]. In psychotic illnesses, clozapine-induced OCS should not be a reason for discontinuation when clear benefits for decreasing psychosis occur. Strategies for managing clozapine-induced OCS include attempting a dose reduction. In one study, clozapine levels were significantly higher in those with OCS versus no OCS, 595.1 ± 364.9 versus 433.5 ± 252.8 ng/mL, *P* = 0.001 [22]. Another management strategy for clozapine-induced OCS is with the use of any serotonergic antidepressant [23]. Due to mild to moderate inhibition of clozapine metabolism caution must be taken if fluoxetine, paroxetine, or clomipramine is selected [24–26]. Fluvoxamine should be avoided as it is a potent inhibitor of clozapine metabolism and may result in toxicity [23]. Other modalities of managing clozapine-induced OCS have been described in single case reports, including but not limited to valproic acid and electroconvulsive therapy [27, 28].

**Figure 1:**
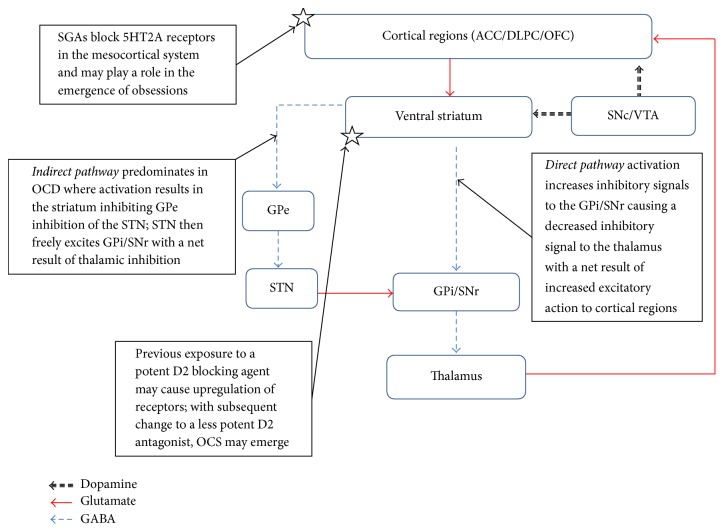
Brief overview of OCD pathophysiology and suspected targets involving antipsychotic-induced OCS/OCD. ACC: anterior cingulate cortex; DLPC: dorsal lateral prefrontal cortex; GPe: globus pallidus pars externalis; GPi: globus pallidus pars internalis; OFC: orbitofrontal cortex; STN: subthalamic nucleus; SNc: substantia nigra pars compacta; SNr: substantia nigra pars reticulata; VTA: ventral tegmental area.

The mechanism of clozapine-induced OCS is poorly understood but may be a result of 5HT2A receptor antagonism in key brain regions associated with OCD, including the anterior cingulate cortex, dorsal lateral prefrontal cortex, and orbitofrontal cortex (OFC) [29, 30] (Figure 1). This explains why other second-generation antipsychotics (SGAs) have been associated with inducing or worsening OCS/OCD [31–33]. First-generation antipsychotics (FGAs) that have primarily dopaminergic actions have not been widely implicated in the emergence OCS/OCD and support the antiserotonergic hypothesis [15, 18]. Interestingly, aripiprazole is a 5HT1A partial agonist and reported to eliminate clozapine-induced OCS [8, 34, 35]. Patients that are exposed to a potent dopamine antagonist before clozapine may be at a greater risk for developing clozapine-induced OCS/OCD. It is thought that clozapine use following FGA-mediated dopamine receptor upregulation in the striatum results in a net reduction of dopamine blockade that potentiates OCS. Recently, Schirmbeck and colleagues conducted the first neuroimaging study to assess the effects of different SGAs on brain regions of the frontostriatothalamocortical circuitry [6]. Specifically, patients prescribed clozapine or olanzapine (the prominent antiserotonergic antipsychotic group) exhibited increased activation in the OFC during response inhibition compared to patients prescribed amisulpride or aripiprazole (the prominent dopaminergic blocking antipsychotic group). Finally, a genetic predisposition may also explain clozapine-induced OCS/OCD, with polymorphisms of various genes (i.e.,* SLC1A1*,* GRIN2B*, and* GRIK2*) linked to this phenomenon [36].

Unique from what is described in the literature is the fact that our patient did not have a primary psychotic disorder but was given clozapine that worsened OCS. Few case reports have described clozapine-induced/worsening OCS in patients without a psychotic disorder and include one adult patient with bipolar disorder and another pediatric patient with OCD [37, 38]. This pediatric case report was similar to ours, in that the patient was initially believed to have a psychotic disorder. A 17-year-old female presenting with intense anxiety, unwanted thoughts of aggression, and hallucinations was diagnosed with schizophrenia. There was no improvement with olanzapine, risperidone, or haloperidol, and when clozapine was initiated the patient's clinical status worsened. A new diagnosis of OCD was considered after the failed treatment attempt with clozapine, and when the medication was discontinued the patient's OCS improved. Fluvoxamine titrated to 300 mg led to remission of OCD in this case. In our case, unfortunately Yale-Brown Obsessive-Compulsive Scale scores were not assessed. However, a temporal relationship existed between the clozapine titrations and the subjective worsening of OCS leading to hospitalization. After clozapine was discontinued, significant OCS improvement was reported by the patient. Using the Naranjo-Adverse-Drug-Reaction-Probability Scale there was a probable relationship between clozapine and OCS worsening [39].

The diagnosis of OCD can be challenging given the heterogeneity of the illness but may include the presence of obsessions that are inaccurately perceived as symptoms associated with a primary psychotic disorder. Clozapine should be avoided in patients with OCD to specifically target OCS as there are limited data to support its use in this setting. Further research is needed to better understand the link between antiserotonergic antipsychotics and OCS.
